# The Hidden Costs of Shifting Away from Flexible Packaging: Implications for Food Prices, Access, and Food Waste Among Low-Income U.S. Households

**DOI:** 10.3390/foods15183188

**Published:** 2026-09-09

**Authors:** Monireh Mahmoudi, Lauren Davis, Natalie Gizoni, Sophia Joseph, Kaitlyn Volpi

**Affiliations:** 1 School of Packaging, College of Agriculture and Natural Resources, Michigan State University, 448 Wilson Road, East Lansing, MI 48824, USA; gizonina@msu.edu (N.G.); josep161@msu.edu (S.J.); volpikai@msu.edu (K.V.); 2 Department of Industrial and Systems Engineering, North Carolina Agricultural and Technical State University, 1121 East Market Street, Greensboro, NC 27411, USA; lbdavis@ncat.edu

**Keywords:** household food waste, food packaging, packaging functionality, food insecurity, food accessibility, low-income households

## Abstract

Packaging plays an important role not only in protecting food and extending shelf life but also in shaping affordability, portability, storage, and the risk of spoilage at the household level. This study examines how flexible plastic and alternative packaging formats differ in retail price and how low-income U.S. households report they would respond to packaging-related changes that could affect food access and food waste. Two complementary approaches were used. First, an online and in-store retail audit compared 57 matched food products sold in flexible plastic and alternative packaging formats. Alternative format products were more expensive on average when prices were normalized by product weight, although the magnitude of the difference varied substantially across products. Second, a survey of 473 U.S. adults with annual household incomes below USD 40,000 examined responses to hypothetical changes in food price, carrying difficulty, storage fit, and spoilage. Ordered logit models showed that food-insecure households reported greater sensitivity to price increases, households facing transportation burdens reported greater difficulty with harder-to-carry packaging, and households with storage constraints reported greater difficulty with packaging that was harder to store. Shopping frequency was the strongest predictor of responses to faster spoilage. Together, the findings indicate that changes in packaging format may create different challenges across households depending on their financial, transportation, storage, and shopping constraints. Because the survey captures reported responses to hypothetical scenarios rather than observed purchasing or food waste behavior, the findings should be interpreted as evidence of household vulnerability to packaging-related changes rather than direct causal effects of packaging transitions or policy.

## 1. Introduction

### 1.1. Motivation

Food loss and waste (FLW) remain a persistent barrier to sustainable and equitable food systems. Target 12.3 of the UN 2030 Agenda calls for halving per-capita global food waste, and coalitions such as Champions 12.3 have pushed this goal onto national policy agendas [1]. Much of the resulting policy attention has focused on production, retail, and distribution stages of the supply chain, yet a substantial share of food waste occurs after food enters the household, where it is shaped by storage capacity, shopping patterns, and the physical properties of the packaging the food arrives in [2,3,4]. Packaging is not incidental to this process: its barrier properties, resealability, portioning, and structural fit with household storage all plausibly influence how much food is used before it spoils.

At the same time, packaging itself is undergoing substantial change. States are increasingly adopting Extended Producer Responsibility (EPR) laws and packaging regulations aimed at reducing environmental waste from non-recyclable materials, including the flexible plastic packaging widely used across the typical U.S. grocery basket. California’s SB 54, for example, requires that single-use plastic packaging be recyclable or compostable by 2032 [1]; comparable initiatives are advancing in New York [5] and New Jersey [6], with additional legislation already enacted in Maine, Oregon, Colorado, and Minnesota [7]. These policies are motivated by upstream environmental goals—reducing non-recyclable material in circulation—rather than by household-level food waste outcomes, and their downstream implications for at-home food waste have not been directly examined.

This gap matters because packaging formats differ in functional properties that are plausibly relevant to food waste: flexible plastic packaging is lightweight and can offer strong barrier protection against moisture and contamination, along with resealability and portioning features [8,9,10], while alternative formats vary widely in these same properties depending on material and design [11]. Whether a shift toward alternative packaging formats increases or decreases at-home food waste is therefore an open, largely untested empirical question, not a foregone conclusion in either direction. This study addresses that question by examining how packaging-format differences relate to price, handling, storage fit, and spoilage risk—examining packaging functionality as one underexamined factor relevant to household food waste risk alongside the storage and shopping-pattern constraints already identified in the food waste literature.

### 1.2. Background

Packaging shapes consumer behavior across the household food lifecycle, influencing purchase, storage, preparation, consumption, and waste. Prior research shows that packaging attributes affect perceived freshness, willingness to pay, and food waste through mechanisms such as portioning, transparency, resealability, and information provision [9,10,11]. Packaging can also influence whether food can be transported conveniently, stored efficiently, protected from deterioration, and consumed before spoilage. However, an important gap remains: little is known about how sustainability-driven packaging policies affect downstream outcomes such as household food affordability, accessibility, and food waste. To our knowledge, no study directly evaluates how policy-induced shifts in packaging formats influence these interrelated household outcomes in the U.S. context.

This gap is critical given extensive evidence that sustainability transitions often generate uneven impacts across populations. Across sectors such as energy, transportation, housing, environmental health, and waste management, disadvantaged communities frequently face higher costs, weaker infrastructure, and reduced access to emerging technologies. For example, energy insecurity remains unevenly distributed across income, race, and housing tenure, while clean-energy investments are concentrated in higher-income areas [12,13,14]. Transportation inequities similarly persist due to long travel distances, limited modal options, and uneven electrification infrastructure [15,16,17]. Housing quality and built-environment conditions further shape energy burdens and exposure risks, while environmental health disparities reflect unequal exposure to air and water pollutants and climate vulnerability [18,19,20,21,22,23,24]. Waste and recycling outcomes also vary with infrastructure capacity and governance, particularly in rural systems [25,26,27].

These patterns motivate the central question of this study: if sustainability policies restrict packaging options, could resulting changes in product pricing, handling requirements, storage conditions, and shelf life generate uneven impacts on food affordability, accessibility, and household food waste? This concern is especially salient in food systems, where access and waste are shaped by structural constraints rather than individual choice alone [28,29,30,31]. The literature identifies several key mechanisms through which households experience food access and manage purchased food: mobility and time constraints, affordability and price dynamics, retail structure and quality, household storage capacity, shopping frequency, and structural disadvantage.

Food accessibility is increasingly conceptualized as a multidimensional construct encompassing geographic proximity, supply adequacy, affordability, acceptability, accommodation, and awareness [32]. Effective access depends not only on the presence of nearby retailers but also on whether households can realistically obtain desired foods given constraints on mobility, time, and resources [33,34,35,36]. Households often bypass nearby stores in favor of options that better align with preferences, prices, or quality, implying that access is defined by activity space rather than administrative boundaries [33,37,38]. Moreover, food access reflects interactions between individual constraints, social supports, and neighborhood-level conditions [39,40]. These same constraints may also affect how frequently households can replenish food supplies and whether purchased foods can be consumed before deterioration.

Mobility and time constraints play a central role. Travel distance, transportation availability, and time schedules strongly influence realized access, with mobility often mediating the relationship between place and outcomes [33,34,37]. Limited mobility can create barriers even in areas with nearby retailers, while temporal constraints such as store hours or transit schedules can render otherwise accessible environments functionally constrained [41]. Rural and urban contexts exhibit distinct challenges, including longer travel distances in rural areas and congestion or transit limitations in urban settings [33,34]. These constraints can also require households to purchase food less frequently or in larger quantities, increasing the importance of packaging that is easy to transport, store, and preserve over time.

Affordability and price dynamics are equally critical. Income, poverty, and food prices strongly determine access to nutritious foods, with low-income households particularly vulnerable to price increases and inflation [34,36,42,43,44,45]. Households with constrained access often face higher prices at smaller retailers, exacerbating food insecurity [34,43]. Financial constraints frequently dominate geographic ones, shaping both store choice and purchasing decisions [42]. They may also influence how households respond when food deteriorates prematurely, because financially constrained households have fewer resources to replace spoiled products or absorb losses from discarded food.

Retail structure and quality further influence access. Store type, product availability, pricing, and competitive dynamics shape purchasing behavior and food accessibility [35,37,43,45,46], while improving retail provision can reduce travel burdens, impacts depend on pricing and product mix [47]. Seasonal variability and limited availability of healthy foods, particularly in rural areas, also constrain access [40]. The packaging formats available within these retail environments may further influence whether foods remain usable after purchase, particularly when households face limited storage capacity or infrequent shopping opportunities.

Household storage conditions and shelf life provide an additional link between packaging, food access, and food waste. Packaging that is bulky, difficult to reseal, poorly suited to refrigerator or pantry space, or less effective at protecting food quality may increase the likelihood that food is damaged, forgotten, displaced, or spoiled before consumption. Conversely, packaging that provides barrier protection, portion control, resealability, and efficient storage fit may support households in preserving food and using a greater share of what they purchase. These functional characteristics are especially important for households with limited refrigerator, freezer, or pantry capacity and for those unable to shop frequently.

Finally, structural inequities and social factors play a cross-cutting role. Food access disparities are linked to race, socioeconomic status, and spatial inequality, with marginalized populations often facing higher time and travel burdens [34,36,48]. Experiences of poor food quality, stress, and historical disinvestment further shape access outcomes [49]. Social supports and policy interventions, including food assistance programs and community initiatives, can mitigate these barriers but remain uneven in availability and effectiveness [38,39,40]. These structural conditions may also shape households’ capacity to prevent food waste by limiting their flexibility to replace spoiled food, change shopping schedules, obtain alternative products, or invest in additional storage.

Taken together, this literature highlights that food accessibility and household food waste are system-level outcomes shaped by interacting constraints related to mobility, affordability, retail conditions, storage capacity, shopping frequency, and structural inequality. However, the role of packaging—and particularly policy-driven changes in packaging formats—remains largely unexplored within this framework. This study addresses this gap by examining how sustainability-oriented packaging transitions may influence food affordability and access while also altering storage fit, shelf life, and the likelihood that purchased food is wasted before consumption.

Throughout this study, the survey outcomes are described as reported responses or response severity, referring to the ordered responses to the four hypothetical scenarios. Household characteristics such as food insecurity, transportation burden, storage constraints, and shopping frequency are treated as potential sources of household vulnerability to these changes. Food accessibility refers to households’ ability to afford and physically obtain food, whereas food waste risk refers only to storage- and spoilage-related conditions that may increase the likelihood of food being discarded. Actual packaging-caused household food waste was not measured. Accordingly, the retail audit is interpreted as a descriptive comparison of currently available products, and the survey is interpreted as evidence of reported responses to hypothetical packaging-related changes rather than observed effects of a policy or packaging transition.

## 2. Materials and Methods

### 2.1. Ethical Statement

All study procedures were reviewed and approved by the Michigan State University Institutional Review Board (IRB# STUDY202500898). An online survey was designed in Qualtrics and administered through Prolific (Start date: 23 March 2026, End date: 23 March 2026). Informed consent was obtained electronically from all participants. The first page of the survey included a detailed consent form outlining the study’s purpose and participant rights. Participants indicated their consent by clicking an “Agree” button before proceeding. The consent form clarified that participation was voluntary and that participants could skip any question, select “I don’t know” or “Other,” or withdraw at any time without penalty.

### 2.2. Experiment Procedure

The functional properties that distinguish packaging formats are directly relevant to household food waste risk, and flexible plastic packaging illustrates this well. Flexible plastic packaging is widely used due to its combined advantages in cost efficiency, consumer convenience, and shelf life performance. Its economic appeal arises from low material use, lightweight structure, and logistics efficiency, as flexible formats reduce production costs relative to rigid alternatives and lower transportation and storage costs through high product-to-package ratios and roll-based delivery [50,51]. Studies further show that flexible packaging can significantly reduce material usage and weight, lowering costs by up to 30%, while also requiring less manufacturing energy than glass or metal [52,53]. Beyond cost, flexible packaging aligns with consumer demand for convenience, offering features such as portability, resealability, easy opening, and portion control that reduce effort and support modern lifestyles [52,54]. Additional research highlights the role of lightweight design, sachet variety, and functional features (e.g., zip-locks, spouts, microwave compatibility) in enhancing usability, freshness, and on-the-go consumption, while disposable formats remain preferred due to minimal post-purchase effort compared to reusable systems [51,53,55,56]. Importantly, flexible plastic packaging also plays a critical role in extending shelf life: multilayer films provide strong oxygen and moisture barriers, mechanical protection, and compatibility with vacuum or modified-atmosphere packaging, helping preserve food quality and reduce spoilage [57,58,59]. Empirical evidence shows that flexible laminates can significantly extend product shelf life by controlling moisture, oxidation, and degradation processes [60], and—notably for a food waste framing—that preventing spoilage of the food itself may yield greater environmental benefit than improvements in packaging recyclability alone [57]. This last point is central to the motivation for the present study: packaging decisions that improve material-level sustainability metrics are not guaranteed to improve, and could plausibly worsen, food-level waste outcomes if shelf life and storage-fit properties are not preserved in alternative formats. Overall, the widespread adoption of flexible plastic packaging reflects its ability to integrate cost efficiency, user convenience, and advanced barrier functionality into a single format [53], which is precisely the functional bundle that alternative formats must replicate if packaging transitions are to avoid shifting waste from the material stream to the food stream.

Guided by this literature, the empirical portion of this study uses two complementary approaches to examine how packaging format relates to food affordability, handling, storage fit, and shelf life—properties that plausibly bear on household food waste. First, Approach 1 uses an online and in-store retail audit to descriptively benchmark prices and packaging formats across comparable food products, documenting how products sold in flexible plastic packaging compare with similar products sold in alternative formats. Second, Approach 2 uses a survey of U.S. consumers, with particular attention to lower-income households, to examine self-reported responses to packaging-related changes in cost, handling, storage fit, and expected shelf life under everyday household constraints.

Approach 1: Online and in-store audit (descriptive price and packaging benchmarking). Two research assistants conducted an online and in-store audit from 20 September 2025 to 20 October 2025. The purpose of this audit was descriptive: to construct a paired dataset of comparable products offered in (i) a flexible plastic packaging format and (ii) an alternative packaging format, and to document how price and packaging format co-occur in current retail offerings, rather than to test a causal effect of a specific policy change. For each observed product, the auditors recorded the following fields for the flexible plastic option: Food category, Food item, Flexible plastic packaging format, Brand, Weight, Store name, Online (Y/N), State, Zipcode, Price. The same fields were recorded for the corresponding alternative packaging option. Data were collected from major retailers (e.g., Walmart, Meijer, Kroger) via online listings (when available) and/or in-store observation. Following food classifications aligned with USDA guidelines, the audit included nine food categories, with up to five food items sampled per category: Breads and Bakery; Snacks; Fresh and Frozen Produce; Dairy; Dry Goods; Cereals; Protein; Infant Foods; and Juice. This category cap keeps the audit a small, purposive, non-random sample of current market offerings rather than a representative sample of the retail landscape, and results from Approach 1 are interpreted accordingly throughout this paper.

**Figure 1:**
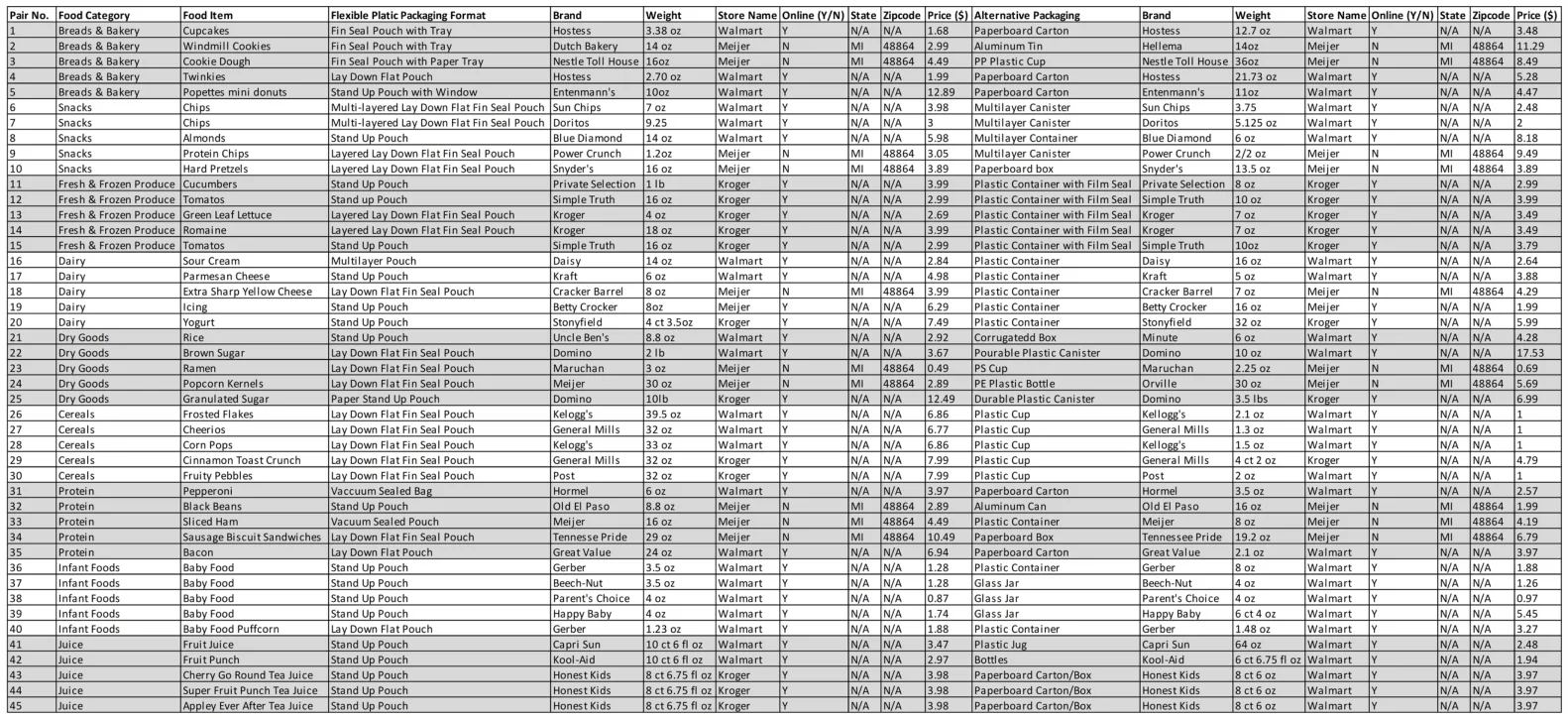
Paired observations from the online and in-store audit (20 September 2025 to 20 October 2025).

Our data collection strategy prioritized brands that offered the same food item—comparable in quality, value, and volume—in both flexible plastic packaging and an alternative packaging format. When a given brand did not offer an equivalent product in a non-flexible plastic (or flexible non-plastic) format, this limitation was documented. In such cases, a comparable brand with a similar market positioning and reputation was identified, provided that it offered a product of equivalent quality, value, and volume in an alternative packaging format. These matched products were then included as paired observations, and their packaging formats and corresponding prices were recorded to describe observed differences in normalized product cost by weight across packaging types. Because paired products can differ in brand, formulation, or market positioning in addition to packaging format, observed price differences reflect the current retail landscape rather than the isolated effect of packaging format alone; this distinction is revisited in Section 4. Figure 1 reports the complete set of paired observations collected during the audit window, including the flexible plastic product and its matched alternative packaging product.

When conducting the online and in-store audit, we observed that for a subset of food items, more than one alternative (non-flexible plastic) packaging format was available for the same product (see Figure S1). Each alternative packaging format was treated as a distinct paired observation with the corresponding flexible plastic package, reflecting separate market offerings rather than duplicate products. This approach resulted in a total of 57 paired observations, each consisting of a product packaged in flexible plastic and a comparable product packaged in an alternative format. Because this comparison relies on a small, purposive, non-random sample and is not evaluated with a formal significance test (see below), it is presented as a research question rather than a formal hypothesis, distinguishing it structurally from the confirmatory hypotheses evaluated in Approach 2 (Research Question 1):

Research Question 1 (RQ1). Do products packaged in flexible plastic and products packaged in alternative formats differ in normalized retail price?

Approach 2: Survey-based assessment of packaging-related waste and accessibility risk. To complement the descriptive price benchmarking in Approach 1, this study employed an online survey to examine how packaging-related changes in handling, storage fit, and shelf life relate to household food waste risk and accessibility under financial and logistical constraints. The survey was administered via Qualtrics and distributed through Prolific to U.S. adults. Eligibility was limited to low-income respondents (annual household income below USD 40,000), English speakers, and individuals with a Prolific approval rate of 97–100% and at least 500 prior submissions. Participants received USD 1.00 upon completion.

The USD 40,000 cutoff was operationalized using Prolific’s available household-income pre-screening bands, which are provided as fixed categories rather than as a continuous income value. We selected the five categories below USD 40,000 (less than USD 10,000; USD 10,000–USD 15,999; USD 16,000–USD 19,999; USD 20,000–USD 29,999; and USD 30,000–USD 39,999). This cutoff balanced the study’s objective of concentrating recruitment on households more likely to be sensitive to packaging-related affordability and accessibility changes with the need to maintain a sufficiently large eligible recruitment pool; applying this income screen together with the study’s other pre-screening criteria yielded 4052 eligible Prolific participants.

The instrument included 20 questions and required approximately 4–5 min to complete. Participation was voluntary and anonymous, with no identifying information collected. Data were stored on password-protected Michigan State University cloud systems and were accessible only to the research team and the Human Research Protection Program. The full instrument is provided in Supplementary Materials.

The survey assessed how packaging-related changes relate to food waste risk and accessibility. After consent, respondents were asked to consider a commonly purchased basic food (e.g., milk, produce, meat, or grains) and indicate how their household would respond to four hypothetical changes: (1) increased price, (2) more difficult carrying (e.g., heavier or bulkier packaging), (3) more difficult storage (e.g., larger or inefficient packaging), and (4) faster spoilage. The latter two scenarios map most directly onto at-home food waste mechanisms, since inadequate storage fit and reduced shelf life are established precursors of household discarding [2,3,4]. Responses for each scenario were recorded on a five-category ordered scale designed to represent a single underlying dimension of increasing behavioral disruption, ranging from no change, through intermediate coping responses (e.g., adjusting quantity, timing, or storage arrangements), to discontinuing purchase of the food altogether. The verbatim response options for each scenario and their assigned ordinal ranks are reported in Table 1. A common ordering rule was applied across the four items: responses were ranked from no change, to adjustments that preserve continued purchase of the food, to substitution, and finally to discontinuing purchase. The ordering therefore reflects increasing departure from the household’s usual purchasing behavior. Because this interpretation is an analytical assumption, the proportional-odds assumption was examined for each ordered logit model and sensitivity analyses were conducted where the assumption was not well supported. This ordering is the basis for treating responses as ordinal outcomes in the modeling approach described below, and proportional-odds diagnostics for each model are reported alongside the results.

To contextualize these outcomes, the survey collected data on storage capacity, food waste, grocery access, food security, and household characteristics. Table 2 documents the construction of each derived predictor variable from its source survey item(s), to support reproducibility. Storage constraints (Q8–Q9) captured refrigerator/freezer and pantry capacity, given their established importance in managing food purchases and preventing waste [2,3]. Self-reported food waste (Q10) was included as a direct household-level waste indicator linked to spoilage and replenishment behavior [4,61]; as this is a single-item, non-validated self-report measure not specific to packaging-driven waste, it is treated as a covariate and its limitations as a waste measure are discussed in the Conclusions. Grocery access (Q5–Q7) included transportation mode, travel time, and shopping frequency, reflecting the role of mobility, temporal constraints, and replenishment flexibility in both realized access and waste risk [62,63,64].

Food security was measured using the USDA six-item module (Q11–Q16), capturing financial and behavioral indicators over the previous 12 months [65]. Standard coding procedures were applied to classify households into high/marginal (0–1), low (2–4), and very low (5–6) food security categories. This variable served as the primary indicator of economic vulnerability.

Finally, the survey included household characteristics associated with food waste and accessibility vulnerability: household size (Q17), presence of children (Q18), participation in assistance programs (Q19), and disability affecting food-related activities (Q20). These factors are closely linked to food insecurity and coping capacity, as larger households, households with children, and those experiencing disability or relying on assistance often face heightened constraints [66,67,68]. Guided by these measures, Approach 2 evaluated the following hypotheses:

 **Hypothesis 1.** *Higher household food insecurity is associated with more severe purchase responses when packaging increases the price of a basic food item.*

 **Hypothesis 2.** *Greater transportation burden is associated with more severe responses when packaging makes a product harder to carry home.*

 **Hypothesis 3.** *More limited household storage capacity is associated with more severe responses when packaging makes a product harder to store at home.*

 **Hypothesis 4.** *Households with less frequent shopping patterns and more constrained storage capacity are more likely to report more severe responses when packaging causes food to spoil more quickly.*

## 3. Results

### 3.1. Results from Approach 1

To ensure comparability across package sizes, all prices were normalized by product weight. Across the 57 paired observations, flexible plastic packaging showed a lower mean price per ounce (mean = USD 0.384, median = USD 0.250, standard deviation [SD] = USD 0.407, interquartile range [IQR] = [USD 0.187, USD 0.427]) than alternative packaging formats (mean = USD 0.508, median = USD 0.363, SD = USD 0.501, IQR = [USD 0.227, USD 0.613]). The median price ratio (alternative ÷ flexible plastic) was 1.19, and the mean ratio was 1.82; the substantial gap between these two summary statistics itself signals that the distribution of price ratios is right-skewed and sensitive to a small number of large values, addressed directly below. Descriptively, then, flexible plastic packaging was associated with a lower normalized price on average across the 57 paired observations in this audit.

Table 3 reports this comparison disaggregated by food category. The price gap is not uniform across categories: Dry Goods and Cereals show the largest average ratios, while Dairy shows flexible plastic as more expensive on average than its alternative format pairings, illustrating that the aggregate pattern reported above does not hold uniformly across all types of food products.

Two pairings show substantially larger price ratios than the rest of the sample: Brown Sugar (ratio = 15.3) and Bacon (ratio = 6.5), both more than three times larger than the next-highest ratio in the audit (Windmill Cookies, ratio = 3.8). As a sensitivity check, excluding these two pairings reduces the mean ratio from 1.82 to 1.49 and the median ratio from 1.19 to 1.16 across the remaining 55 pairings (flexible plastic mean = USD 0.391/oz, alternative mean = USD 0.460/oz), indicating that the central tendency of the comparison is directionally robust to these two extreme values, though its magnitude is meaningfully smaller once they are excluded.

A separate consideration concerns the independence of observations within the audit. Of the 57 paired observations, 13 correspond to a base flexible plastic product (e.g., Cheerios, Frosted Flakes, Brown Sugar, Rice, Parmesan Cheese) that was paired with two different alternative format products, accounting for 26 of the 57 pairings (46%); the remaining 31 pairings each correspond to a base product used only once. When the audit is collapsed to one observation per unique base product (n=44, averaging across a product’s alternative format matches where more than one exists), the mean price ratio is 1.67 and the median ratio is 1.17—close to the full-sample values above, indicating that the central tendency is not substantially driven by repeated products. As a secondary robustness note only, a paired comparison is directionally similar but loses conventional statistical significance once collapsed to unique products (full sample: paired t=2.22, p=0.031; collapsed to unique products: paired t=1.57, p=0.124), indicating that confidence in a non-zero average difference is more sensitive to this clustering structure than the central tendency itself. Given the audit’s small, non-random, purposive sample, this comparison is reported descriptively throughout this manuscript rather than as a hypothesis test, consistent with Research Question 1 as stated in Section 2.

This descriptive difference should be interpreted with two caveats. First, because the audit pairs distinct products rather than tracking the same product across a packaging change, the observed price difference reflects the current retail landscape—including brand, formulation, and market-positioning differences between paired products—rather than an isolated, causal effect of packaging format on price. Second, as shown above, the magnitude of the aggregate difference is sensitive to a small number of paired observations with unusually large price ratios (brown sugar and bacon) and to the repeated use of some base products across multiple pairings, both quantified above rather than left as a general caveat.

### 3.2. Results from Approach 2

Sample and household characteristics: A target of 500 completed responses was established before data collection. The target was selected to exceed a simple conservative survey sample-size calculation. For a proportion estimated with 95% confidence, 5% precision, and the most conservative assumption of p=0.50, the required sample is $n=\left(1.96^{2}\times 0.50\times 0.50\right)/\left(0.05^{2}\right)=384.16$, or approximately 385 respondents. Targeting 500 therefore provided a margin for incomplete or excluded responses while remaining above this minimum. After exclusions for missing study variables and incomplete or quality-screened responses, the final analytic sample was 473. At n=473, the corresponding maximum 95% margin of error for a proportion near 50% is approximately 4.5%. Sample demographic characteristics (age, sex, ethnicity, household income, employment, and student status) and geographic distribution are reported in Table 4.

Because the Qualtrics export did not retain the Prolific participant identifier, Prolific demographic records were linked to survey records using precise timestamps recorded on the two platforms. Matching was constrained to be one-to-one: each Qualtrics record was paired with the nearest still-unmatched Prolific record by timestamp, after which both records were removed from further matching. Of the 473 analytic records, 462 (98%) matched within seconds (median gap approximately 2 s), and the remaining 11 matched within several minutes. Separately, 11 respondents whose Prolific status was subsequently recorded as RETURNED or TIMED-OUT were retained in the analytic sample because each had a submitted Qualtrics record containing the study variables required for analysis and passed the same survey data-quality screening applied to all respondents. Inclusion was therefore based on the completed survey record and the study’s analytic and data-quality criteria rather than on the later Prolific status field. Demographic information for these 11 records was unavailable in the Prolific demographic export, so they were excluded only from the demographic characteristics table.

Using the USDA six-item food security module, 236 respondents (49.9%) were classified as having high or marginal food security, 101 (21.4%) as having low food security, and 136 (28.8%) as having very low food security. Thus, slightly more than half of the sample met the criteria for food insecurity (low or very low food security). Sample characteristics are summarized in Table 5. Most respondents reported using their own car to shop for groceries (63.9%), followed by someone else driving them (14.4%) and walking (7.8%). Refrigerator/freezer storage was reported as “enough” by 52.2% of respondents, while 25.4% reported it as “somewhat limited” and 6.3% as “very limited.” Pantry or cabinet space was reported as “enough” by 44.8% of respondents, with 28.5% reporting it as “somewhat limited” and 7.8% as “very limited.”

Food security groups did not differ significantly in household size, primary transportation mode, travel time to the main grocery store, or grocery shopping frequency. By contrast, food security status was significantly associated with refrigerator/freezer space, pantry space, current food waste, presence of children in the household, assistance participation, and disability status (Table 5). In general, lower food security was associated with tighter household storage conditions and higher prevalence of assistance participation and disability.

**Figure 2:**
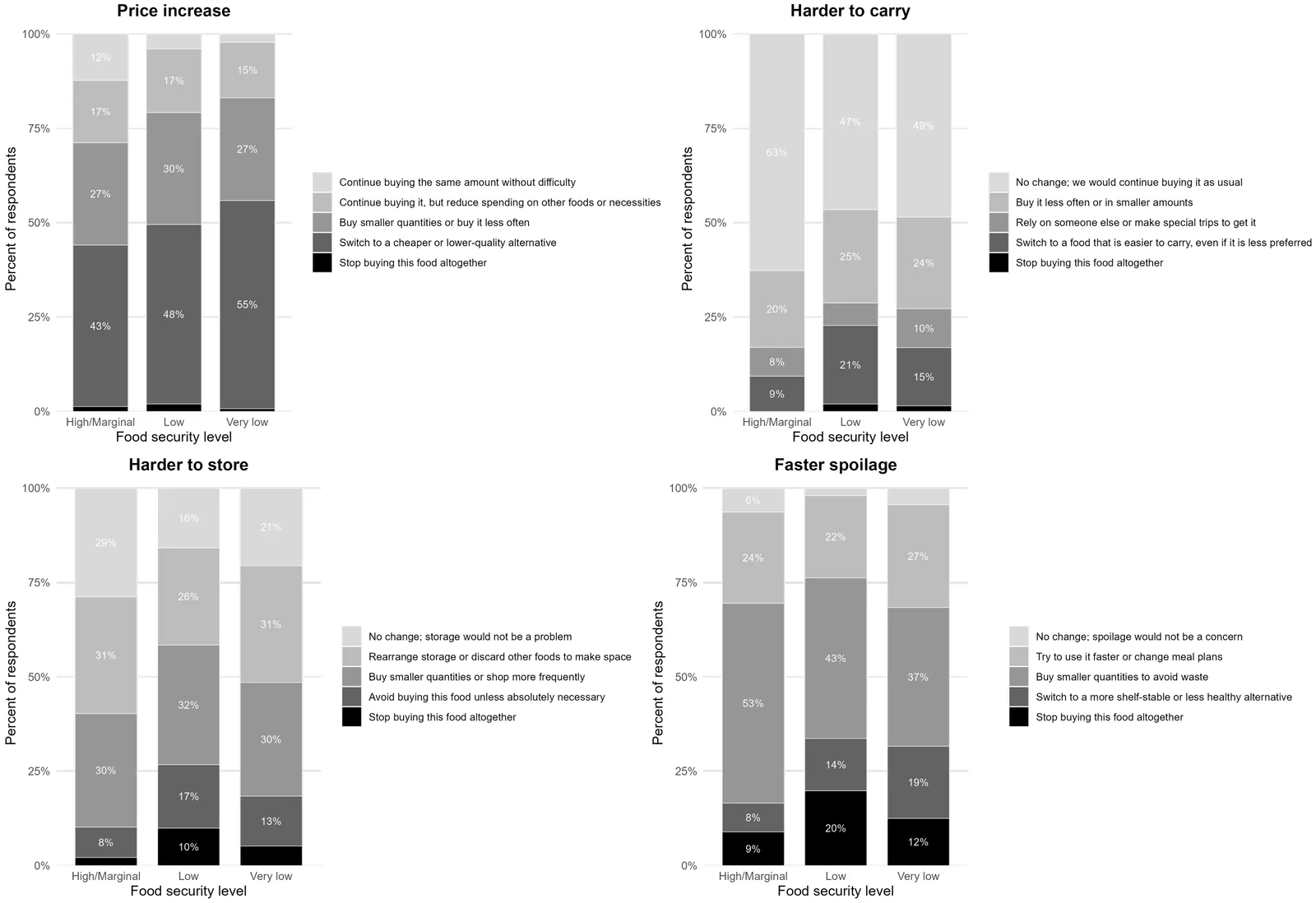
Distribution of responses to four packaging-change scenarios by household food security status. Panels show responses to packaging changes that make a food item more expensive, harder to carry, harder to store, or more likely to spoil quickly. Food security status is classified using the USDA six-item food security module.

Descriptive responses to packaging changes: Figure 2 displays response distributions for the four packaging-change scenarios by food security status. Full percentages for each response category are provided in Table S1. The storage and spoilage scenarios are of particular interest for food waste, since several of their response options (e.g., discarding other foods to make space, buying smaller quantities to avoid waste) describe waste-relevant behavior directly, rather than only affordability or access behavior.

Price-related packaging changes generated the strongest economic adjustment responses. Overall, 47.4% of respondents indicated that if packaging made a regularly purchased basic food more expensive, they would switch to a cheaper or lower-quality alternative, and an additional 27.7% reported that they would buy smaller quantities or buy the food less often. Only 7.6% indicated that they would continue buying the same amount without difficulty.

Packaging changes that reduced carrying convenience also altered reported food-access behavior, although less dramatically. For this scenario, 55.2% reported no change, but 22.4% indicated that they would buy the food less often or in smaller amounts, 13.5% would switch to a food that was easier to carry, and 8.0% would rely on someone else or make special trips to obtain it.

Storage-related packaging changes produced substantial reported adjustments. When packaging was described as harder to store at home, 30.4% reported that they would buy smaller quantities or shop more frequently, 29.8% would rearrange storage or discard other foods to make space, and 11.4% would avoid buying the food unless absolutely necessary. Only 23.7% reported that storage would not be a problem.

Spoilage-related packaging changes were especially disruptive. When packaging was described as causing food to spoil faster, 46.1% reported that they would buy smaller quantities to avoid waste, 24.5% would try to use the food faster or change meal plans, 12.3% would switch to a more shelf-stable or less healthy alternative, and 12.3% would stop buying the food altogether. Fewer than 5% reported that faster spoilage would not be a concern.

Across all four scenarios, the descriptive distributions in Figure 2 indicate a consistent shift toward more severe responses among food-insecure households, particularly for price increases, storage difficulty, and faster spoilage.

Bivariate differences by food security status: Bivariate tests confirmed that packaging-related responses differed significantly across food security groups. Pearson chi-square tests showed significant associations between food security status and responses to price increase (p=0.024), harder-to-carry packaging (p=0.016), harder-to-store packaging (p=0.007), and faster spoilage (p=0.001). Because response categories were ordered, nonparametric Kruskal–Wallis tests were also estimated and yielded the same substantive conclusion: more food-insecure households reported more severe responses to all four packaging scenarios.

For descriptive purposes, responses to Q1–Q4 were also summed to form a Cumulative Response Score. Because the four scenarios represent distinct mechanisms and were not designed as a psychometric scale, the summed score is treated only as a secondary descriptive summary; conclusions are based primarily on the four scenario-specific models. Mean score values were 9.80 for households with high or marginal food security, 11.4 for households with low food security, and 10.9 for households with very low food security A Kruskal–Wallis test showed significant differences across the three food security groups (p<0.001), indicating higher overall reported response severity among food-insecure households.

Spearman correlations among the key constructs are reported in Table S2. Correlations among the explanatory variables were modest (all |r|≤0.19), and the correlation between the Cumulative Response Score and its strongest predictor, food insecurity, was r=0.21, indicating that multicollinearity is unlikely to distort the multivariate estimates reported below.

Analytic structure: The four scenario-specific ordered logit models are the primary analyses used to evaluate the survey hypotheses. The proportional-odds diagnostics and sensitivity analyses are used to assess model robustness, while analyses of the Cumulative Response Score are retained only as secondary descriptive analyses.

Multivariable models of packaging-related response severity: To examine the four packaging scenarios separately, ordered logit models were estimated for price increase, harder-to-carry packaging, harder-to-store packaging, and faster spoilage. The main results are presented in Table 6.

For the price increase scenario, higher food insecurity was associated with significantly more severe responses. Holding transportation burden, storage constraints, food waste, shopping frequency, household size, children, assistance participation, and disability constant, food-insecure households were more likely to report reducing purchases, switching to lower-quality alternatives, or discontinuing purchase when packaging increased the price of a basic food item. None of the other explanatory variables reached statistical significance in this model, suggesting that the price channel was driven primarily by household food insecurity.

For the harder-to-carry scenario, transportation and physical constraints played a stronger role than food insecurity alone. Greater transportation burden was associated with more severe reported responses, as were tighter storage constraints, greater household food waste, and disability. In contrast, the food insecurity score was positive but not statistically significant at conventional levels. These results suggest that reported severity for packaging that is harder to carry is most strongly associated with mobility and logistical constraints rather than economic vulnerability alone.

For the harder-to-store scenario, storage constraints were the dominant predictor. Households with more limited refrigerator/freezer and pantry space were significantly more likely to report more severe responses to harder-to-store packaging. In addition, households with children reported more severe responses, whereas larger households reported somewhat less severe responses. Food insecurity was positive but not statistically significant in this model after accounting for the other covariates.

For the faster spoilage scenario, shopping frequency emerged as the clearest predictor. Households that shopped less frequently or on a less regular schedule were significantly more likely to report more severe spoilage-related responses. Storage constraints were positive but did not reach statistical significance at conventional thresholds in the main spoilage model, and the corresponding hypothesis (Hypothesis 4) is accordingly treated as only partially supported: the shopping-frequency component is well supported, while the storage-constraint component is not. Food insecurity was also not statistically significant in this model. This pattern indicates that the ability to replenish food regularly may be especially important when packaging reduces usable shelf life.

Taken together, the ordered logit models suggest that reported severity of packaging-related responses operates through distinct mechanisms rather than a single common pathway. Food insecurity was most clearly linked to reported severity in the price scenario, transportation burden was most relevant for the carrying scenario, and storage limitations were most relevant for the storage scenario—the scenario most directly tied to at-home food waste risk. Reported severity in the spoilage scenario, also directly relevant to food waste, appeared to be driven more by shopping patterns than by food insecurity alone.

Proportional-odds diagnostics and sensitivity analyses: The proportional-odds assumption was evaluated for each ordered logit model by comparing it with an unconstrained multinomial logit model containing the same predictors. A *p*-value below 0.05 was treated as evidence that the proportional-odds assumption was not supported. Results are shown in Table 7. The assumption was supported for the carrying and storage models but not for the price and spoilage models.

As a sensitivity analysis, each scenario was also estimated using a binary logistic specification. The substantive direction of the main predictors remained consistent with the ordered models. For the spoilage scenario, where the proportional-odds assumption was not supported, a severe-response specification (switching to a shelf-stable alternative or discontinuing purchase versus all less severe responses) continued to identify shopping frequency as a significant predictor (OR =1.47, p<0.001). These checks support the main interpretation while indicating that the ordered-logit coefficient for the spoilage scenario should be interpreted with caution.

Key results from the binary-logistic sensitivity specifications for the price and spoilage outcomes are reported in Supplementary Table S3.

Secondary summary model and supplementary analyses: As a secondary, exploratory summary analysis, the Cumulative Response Score was regressed on the same set of explanatory variables using ordinary least squares (Table 8). Consistent with the channel-specific models, higher food insecurity and greater storage constraints were both associated with a higher overall score. Disability was also positively associated with the score, while larger household size was negatively associated with it. Children were positively associated with the score at a marginal level of significance.

Interaction models were estimated to test whether packaging burdens were amplified when food insecurity was combined with transportation or storage constraints. Specifically, we tested a food insecurity × transportation burden interaction in the carrying model and a food insecurity × storage constraint interaction in the spoilage model. Neither interaction term was statistically significant (Table S4), indicating that the main effects of food insecurity and logistical constraints were more important than their multiplicative combination in this sample.

Overall, the results presented in Table 9 indicate that reported severity of packaging-related responses varies across low-income households through multiple, largely distinct pathways. Reported price-related severity was most closely tied to economic vulnerability, reported carrying-related severity was concentrated among households with transportation and mobility constraints, reported storage-related severity was concentrated among households with limited home storage, and reported spoilage-related severity was especially salient for households with less frequent shopping patterns—the latter two being the pathways most directly relevant to at-home food waste. Across the analyses, food insecurity remained an important predictor of overall reported vulnerability, but the data also show that reported severity is not purely a function of economic constraint: it is shaped by a broader set of household logistical constraints, including several with direct bearing on food waste risk.

## 4. Discussion

Across both the retail audit and the household survey, this study finds that packaging format is associated with differences in price, handling, storage fit, and reported spoilage risk—and that these four dimensions operate through largely distinct pathways rather than a single common mechanism. Because the storage and spoilage pathways bear most directly on at-home food waste, this pattern is relevant to understanding packaging functionality as a candidate, household-level correlate of anticipated vulnerability to food waste-relevant disruptions—not a demonstrated driver of food waste itself, consistent with the distinction between reported response severity, inferred food waste risk, and directly measured food waste set out in Section 1—alongside packaging’s more commonly studied role in affordability.

The audit results are directionally inconsistent with a null difference in price between packaging formats (Research Question 1); flexible plastic packaging shows a lower normalized price on average than alternative formats. This descriptive difference is worth interpreting carefully. Because the audit compares distinct products offered side by side in the current market rather than the same product before and after a packaging change, several factors besides packaging format itself could plausibly contribute to the observed price gap—for example, consumer willingness to pay a premium for products marketed as sustainable or reusable, differences in brand positioning, or category-specific production costs unrelated to packaging [50,51]. The finding is best read as evidence that flexible plastic and alternative formats currently occupy different price points in the market, not as a causal estimate of what would happen to a given product’s price if its packaging were changed by policy. This pattern is also sensitive to a small number of high-ratio product pairings and to the repeated use of some base products across multiple pairings in the audit, both discussed in the Results. Within this observed price landscape, food-insecure households reported significantly more severe responses to price increases (Hypothesis 1), consistent with a substantial body of evidence that low-income households are disproportionately sensitive to food price changes generally [34,42,44].

Handling burden followed a different pattern. Households facing greater transportation burden reported more severe responses to packaging that is harder to carry (Hypothesis 2), and this association held independently of food insecurity: the interaction between food insecurity and transportation burden was not statistically significant. Mobility constraints and economic constraints therefore appear to operate as separate, additive sources of vulnerability to packaging-related handling burden in this sample, rather than compounding one another multiplicatively—consistent with prior work suggesting that mobility and affordability are related but distinguishable dimensions of food access [33,42].

The storage and spoilage results speak most directly to food waste risk. Storage constraints were the dominant predictor of reported severity for packaging that is harder to store (Hypothesis 3), and households with children reported more severe responses as well, consistent with prior evidence that in-home storage capacity is a consistent, if underexamined, determinant of household food waste [2,3]. Shopping frequency, in turn, was the strongest predictor of reported severity for faster-spoiling packaging, while the hypothesized role of storage constraints in this same model was positive but not statistically significant at conventional thresholds; Hypothesis 4 is accordingly treated as only partially supported, suggesting that households with less flexibility to replenish food supplies are particularly exposed to shelf life reductions—a pattern consistent with the broader food waste literature linking replenishment patterns and economic constraint to waste behavior, including the well-documented paradox that resource-constrained households can experience elevated waste risk despite strong incentives to minimize it [4,61]. Notably, food insecurity was not a significant independent predictor in either the storage or spoilage models once these logistical constraints were accounted for, indicating that storage capacity and shopping frequency capture a meaningful dimension of food waste vulnerability that is not fully reducible to economic status alone.

Taken together, these patterns point to a genuine trade-off space in packaging design that is easy to overlook if packaging is evaluated on a single dimension at a time. A package can, in principle, be inexpensive but poorly suited to constrained storage; protective of shelf life but costly to produce; or easy to carry but bulky to store. Because this study finds that price, handling, storage fit, and spoilage risk are driven by substantially different household characteristics, a packaging format that performs well on one dimension offers no guarantee of performing well on another. This has a direct implication for food waste prevention: packaging changes motivated primarily by upstream environmental goals—such as recyclability under EPR frameworks—should be evaluated for their storage-fit and shelf life properties specifically, not assumed to be neutral with respect to at-home food waste simply because they address a different (material-level) form of waste.

Two interpretive limitations bear directly on how these findings should be used. First, the survey measures households’ anticipated or reported responses to hypothetical packaging-change scenarios, not observed purchasing or waste behavior; a household that reports it would discard other food to make room for a harder-to-store item may or may not do so in practice. Establishing behavioral and waste outcomes with more certainty would require complementary designs such as kitchen diary studies, retail scanner data, or waste-composition audits following an actual packaging change. Second, several scenario descriptions (e.g., an undefined magnitude of price increase, or a single example product per scenario) leave room for respondents to interpret the severity of the underlying change differently; anchoring future instruments to specific, audited packaging and price differentials would sharpen this measurement.

From a practical standpoint, these findings suggest that food waste prevention strategies operating at the household level—consumer education, portioning guidance, or storage-capacity support—are likely to be most effective when paired with attention to the functional design of the packaging itself, particularly for households with limited storage capacity or infrequent shopping schedules. Manufacturers and retailers redesigning packaging to meet recyclability or material-reduction goals have an opportunity to preserve or improve resealability, portioning, and barrier-protection features in the process, which the present results suggest are directly relevant to household-level waste outcomes for financially and logistically constrained households.

Consistent with the pattern of results above, the audit findings are descriptively inconsistent with a null price difference between packaging formats (Research Question 1), while the survey findings are broadly consistent with Hypotheses 1–3 and partially consistent with Hypothesis 4; interaction effects between food insecurity and logistical constraints, examined for the carrying and spoilage scenarios, were not statistically significant.

## 5. Conclusions

This study examines packaging format as a candidate, household-level correlate of anticipated vulnerability to food waste risk, using a descriptive retail audit alongside a survey of low-income U.S. households. The retail audit finds that flexible plastic and alternative packaging formats differ in normalized retail price in the current market, while the survey finds that households’ reported severity of response to packaging-related change varies systematically with food insecurity, transportation burden, storage capacity, and shopping frequency, depending on the type of change involved.

These patterns are not uniform across households or across pathways. Reported price-related severity was most closely associated with food insecurity, reported handling-related severity with transportation burden, reported storage-related severity with in-home storage capacity, and reported spoilage-related severity with shopping frequency, though the hypothesized additional role of storage constraints in the spoilage pathway was not supported at conventional significance thresholds (Hypothesis 4, partially supported; Table 10). The latter two pathways—storage fit and shelf life risk—connect most directly to household food waste risk, positioning packaging functionality as an underexamined candidate factor in food waste prevention alongside the more commonly studied roles of consumer behavior and food storage practices.

The broader implication of this work is that packaging change—whether driven by sustainability regulation, cost pressures, or market trends—should be evaluated not only for its material and environmental properties but also for its functional fit with household storage capacity and shopping patterns. As packaging regulations such as Extended Producer Responsibility laws continue to expand, assessing how resulting packaging formats perform on storage fit and shelf life protection, and not price alone, is directly relevant to food waste prevention goals.

Several limitations should inform how these findings are interpreted and extended. The retail audit is a small, purposive, non-random sample of paired products rather than a representative or longitudinal sample, and its price comparisons reflect the current retail landscape rather than an isolated, causal effect of packaging format; the descriptive price difference is sensitive to two high-ratio product pairings and to the repeated use of 13 base products across multiple pairings, and a paired significance test (reported only as a secondary robustness note, not as evidence for the main descriptive comparison) loses conventional significance once this repeated-product structure is addressed (Results). The survey captures households’ reported or anticipated responses to hypothetical scenarios rather than observed purchasing or waste behavior, and several scenario descriptions (e.g., an unspecified price-increase magnitude) leave room for interpretive variation across respondents; future instruments would benefit from scenarios anchored to specific audited price and packaging differentials, neutrally worded response options, and explicit attention-check items. Relatedly, the only direct measure of household food waste in this study (Q10) is a single self-reported item that has not been independently validated and was not designed to isolate packaging-driven waste specifically from other sources of household food waste; it is used here as a covariate, not as an outcome, and should not be read as a validated food waste scale. The sample is restricted to low-income U.S. households, which is appropriate for identifying vulnerability but limits generalizability to higher-income populations; The ordered logit specification showed support for the proportional-odds assumption in the storage model, borderline support in the carrying model, and evidence of violation in the price and—most notably—spoilage models; robustness checks (binary-outcome and multinomial specifications) indicate that the substantive conclusions are not artifacts of this specification choice, though the spoilage model’s single summary odds ratio likely understates the concentration of the shopping-frequency effect among the most severe, food waste-relevant responses. Finally, this study relies on self-reported food waste and does not directly measure waste quantities; pairing survey measures with kitchen diary or waste-audit data is a natural next step for establishing packaging’s role in food waste more directly.

The fixed household-income cutoff also does not adjust for household size or geographic variation in cost of living; consequently, households with similar incomes may face substantially different levels of material constraint, and some households above the cutoff may experience hardship comparable to households included in the sample. This limitation should be considered when interpreting the scope and generalizability of the findings.

Overall, this study positions packaging functionality—not packaging material alone—as a relevant and underexamined candidate factor associated with household food waste risk, operating through storage fit and replenishment-related pathways that are largely distinct from affordability. This association is based on households’ reported and anticipated responses to hypothetical scenarios, not on observed purchasing or waste behavior, and should be read accordingly. Food waste prevention strategies, and packaging policy more broadly, stand to benefit from evaluating packaging changes against this fuller set of household-level functional dimensions.

## Figures and Tables

**Table 1:** Verbatim response options for Q1–Q4, with assigned ordinal rank.

Rank	Q1: Price	Q2: Carry	Q3: Storage	Q4: Spoilage
1 (no change)	Continue buying the same amount without difficulty	No change; we would continue buying it as usual	No change; storage would not be a problem	No change; spoilage would not be a concern
2	Continue buying it, but reduce spending on other foods or necessities	Buy it less often or in smaller amounts	Rearrange storage or discard other foods to make space	Try to use it faster or change meal plans
3	Buy smaller quantities or buy it less often	Rely on someone else or make special trips to get it	Buy smaller quantities or shop more frequently	Buy smaller quantities to avoid waste
4	Switch to a cheaper or lower-quality alternative	Switch to a food that is easier to carry, even if it is less preferred	Avoid buying this food unless absolutely necessary	Switch to a more shelf-stable or less healthy alternative
5 (most disruptive)	Stop buying this food altogether	Stop buying this food altogether	Stop buying this food altogether	Stop buying this food altogether

Notes: Ranks were assigned using a single rule applied consistently across all four items: increasing departure from baseline purchasing behavior, from no change, through adjustments that preserve continued purchase of the same food, through product substitution, to discontinuation.

**Table 2:** Construction of derived predictor variables.

Variable	Source Item(s)	Coding Rule	Type
Transportation burden	Q5	1 if primary transport mode is not “My own car”; 0 if “My own car”	Binary
Near store	Q6	1 if travel time to main grocery store is “Less than 10 min”; 0 otherwise; used descriptively but not entered as a predictor in the regression models	Binary
Shopping frequency	Q7	Ordered 1 (More than once per week) to 5 (No regular schedule), i.e., higher values indicate less frequent shopping	Ordinal (1–5)
Storage constrained	Q8, Q9	1 if refrigerator/freezer space (Q8) *or* pantry space (Q9) is reported as “Somewhat limited” or “Very limited”; 0 otherwise	Binary
Household food waste	Q10	1 if “A moderate amount” or “A large amount”; 0 if “Almost none”; single-item self-report, see Conclusions for limitations	Binary
Food insecurity score	Q11–Q16	USDA six-item module; each affirmative response (“sometimes/often true,” “yes,” or an affirmative frequency response) scores 1; summed to a 0–6 raw score	Continuous (0–6)
Food security group	Q11–Q16	Raw score 0–1 = High/Marginal; 2–4 = Low; 5–6 = Very Low, per standard USDA classification	Categorical (3 levels)
Household size	Q17	Numeric household size; “5+” recoded to 5	Continuous
Children in household	Q18	1 if “Yes”; 0 if “No”	Binary
Assistance participation	Q19	1 if any program selected (SNAP, WIC, Medicaid, school meals, food pantry); 0 if “None of the above”	Binary
Disability	Q20	1 if “Yes”; 0 if “No”	Binary

**Table 3:** Price ratio (alternative ÷ flexible plastic) by food category.

Category	*n* Pairings	Mean Price/oz (flex)	Mean Price/oz (alt)	Mean Pairwise Ratio	Median Pairwise Ratio
Breads & Bakery	5	USD 0.603	USD 0.393	1.16	0.55
Cereals	10	USD 0.218	USD 0.480	2.22	2.20
Dairy	6	USD 0.614	USD 0.403	0.69	0.74
Dry Goods	7	USD 0.176	USD 0.637	4.31	2.15
Fresh & Frozen Produce	5	USD 0.303	USD 0.430	1.73	2.03
Infant Foods	8	USD 0.483	USD 0.497	0.87	0.85
Juice	6	USD 0.064	USD 0.078	1.22	1.12
Protein	5	USD 0.384	USD 0.725	2.17	1.11
Snacks	5	USD 0.821	USD 1.015	1.54	1.19

Notes: Mean price/oz (flex) and Mean price/oz (alt) are the arithmetic means of the normalized prices for the flexible plastic and alternative format products, respectively, within each food category. For each matched product pair, the price ratio was calculated as the alternative format price per ounce divided by the corresponding flexible plastic price per ounce. The Mean pairwise ratio is the arithmetic mean of these individual pairwise ratios within the category, whereas the Median pairwise ratio is the median of those ratios. For example, if five matched product pairs had individual price ratios of 0.40, 0.50, 0.55, 0.70, and 3.65, the Mean pairwise ratio would be (0.40+0.50+0.55+0.70+3.65)/5=1.16, whereas the Median pairwise ratio would be 0.55. Thus, the Mean pairwise ratio is not necessarily equal to Mean price/oz (alt) divided by Mean price/oz (flex).

**Table 4:** Sample demographic characteristics.

Characteristic	Category	n (%) or Mean (SD)
Age (n = 462)	Mean (SD)	44.6 (14.4)
	Median [range]	43 [18–82]
Sex (n = 462)	Female	276 (59.7%)
	Male	185 (40.0%)
	Prefer not to say	1 (0.2%)
Ethnicity, simplified (n = 461)	White	332 (72.0%)
	Black	68 (14.8%)
	Mixed	30 (6.5%)
	Asian	20 (4.3%)
	Other	11 (2.4%)
Household income, USD (n = 462)	Less than USD 10,000	106 (22.9%)
	USD 10,000–USD 15,999	78 (16.9%)
	USD 16,000–USD 19,999	51 (11.0%)
	USD 20,000–USD 29,999	90 (19.5%)
	USD 30,000–USD 39,999	137 (29.6%)
Census region (n = 473)	South	214 (45.2%)
	Midwest	105 (22.2%)
	West	91 (19.2%)
	Northeast	63 (13.3%)

Notes: Demographic characteristics were obtained from Prolific participant records and merged with the cleaned survey sample by timestamp, since the survey export did not retain a direct participant identifier. Demographic reporting excludes 11 respondents whose Prolific submission status was RETURNED or TIMED-OUT (retained in the main analytic sample, n = 473); a small number of participants who revoked consent for demographic data use are also excluded from the relevant fields, yielding the smaller n shown per row. Geographic region was derived from self-reported ZIP code (Q0) for the full analytic sample and is based on U.S. Census Bureau region definitions; respondents were located in 49 states. See the text above for the one-to-one timestamp-matching procedure and the analytic-sample inclusion rationale for respondents with RETURNED or TIMED-OUT Prolific status.

**Table 5:** Sample characteristics by food security status.

	Overall	High/Marginal	Low	Very Low	*p*-Value
N	473	236	101	136	
Food insecurity raw score, mean (SD)	2.34 (2.4)	0.23 (0.42)	2.74 (0.82)	5.69 (0.46)	<0.001
Household size, mean (SD)	2.2 (1.21)	2.15 (1.17)	2.19 (1.22)	2.29 (1.27)	0.5665
Own car for groceries	302 (63.85%)	164 (69.49%)	64 (63.37%)	74 (54.41%)	0.1347
Less than 10 min to main store	229 (48.41%)	120 (50.85%)	48 (47.52%)	61 (44.85%)	0.2760
Weekly grocery shopping	223 (47.15%)	123 (52.12%)	46 (45.54%)	54 (39.71%)	0.1337
Refrigerator space somewhat/very limited	150 (31.71%)	54 (22.88%)	36 (35.64%)	60 (44.12%)	0.0007
Pantry space somewhat/very limited	172 (36.36%)	75 (31.78%)	33 (32.67%)	64 (47.06%)	0.0084
Moderate or large household food waste	121 (25.59%)	49 (20.76%)	29 (28.71%)	43 (31.62%)	0.0486
Children in household	87 (18.39%)	30 (12.71%)	23 (22.77%)	34 (25.00%)	0.0057
Any assistance participation	241 (50.95%)	94 (39.83%)	52 (51.49%)	95 (69.85%)	<0.001
Disability affecting food shopping/cooking/storage	89 (18.82%)	30 (12.71%)	20 (19.80%)	39 (28.68%)	0.0007

Notes: Food security categories were derived from the USDA six-item food security module. *p*-values below 0.001 are reported as “<0.001” rather than as a rounded decimal. Categorical variables (all rows except food insecurity score and household size) were compared across the three food security groups using Pearson’s chi-square test of independence on the full, unrecoded set of response categories for each variable (e.g., all five shopping-frequency categories, not a binary collapse); degrees of freedom therefore vary by variable. The two continuous variables (food insecurity raw score, household size) were compared using one-way ANOVA across the three groups.

**Table 6:** Ordered logit models of reported severity of packaging-related responses.

	Q1 Price Increase	Q2 Harder to Carry	Q3 Harder to Store	Q4 Faster Spoilage
Food insecurity raw score	1.10 ** [1.02, 1.19]	1.07 * [0.99, 1.16]	1.05 [0.98, 1.13]	1.05 [0.98, 1.14]
Transportation burden	0.97 [0.82, 1.14]	1.23 ** [1.04, 1.45]	1.07 [0.92, 1.26]	0.97 [0.82, 1.13]
Storage constraints	1.09 [0.96, 1.24]	1.16 ** [1.02, 1.32]	1.31 *** [1.16, 1.48]	1.12 * [0.99, 1.27]
Current food waste	1.11 [0.76, 1.62]	1.63 ** [1.11, 2.39]	1.17 [0.82, 1.67]	1.04 [0.71, 1.51]
Shopping frequency	1.07 [0.89, 1.29]	0.91 [0.76, 1.10]	0.98 [0.82, 1.17]	1.29 *** [1.07, 1.55]
Household size	0.96 [0.81, 1.13]	0.85 * [0.71, 1.02]	0.79 *** [0.67, 0.93]	0.92 [0.78, 1.09]
Children in household	1.02 [0.60, 1.73]	1.23 [0.70, 2.15]	1.71 ** [1.03, 2.84]	1.37 [0.80, 2.35]
Any assistance participation	0.89 [0.62, 1.28]	0.81 [0.55, 1.20]	0.99 [0.69, 1.42]	0.89 [0.62, 1.29]
Disability	1.39 [0.88, 2.23]	1.68 ** [1.07, 2.63]	1.34 [0.87, 2.06]	1.46 [0.93, 2.30]
Observations	473	473	473	473

Notes: Entries are odds ratios with 95% confidence intervals from ordered logit models. Odds ratios greater than 1 indicate a higher likelihood of reporting a more severe packaging-related response. * p<0.10, ** p<0.05, *** p<0.01.

**Table 7:** Proportional-odds likelihood-ratio test (ordered logit vs. multinomial logit).

Scenario	LR Statistic	df	*p*-Value	Proportional-Odds Assumption
Q1 Price	36.74	24	0.047	Borderline violation
Q2 Carry	36.22	24	0.052	Borderline supported
Q3 Storage	24.74	24	0.420	Supported
Q4 Spoilage	38.31	24	0.032	Violation

Notes: LR $=2\times ({\log-\mathrm{likelihood}}_{\mathrm{multinomial}}-{\log-\mathrm{likelihood}}_{\mathrm{ordered}\;\mathrm{logit}})$; df is the difference in the number of estimated parameters between the two models. p<0.05 is treated as evidence against the proportional-odds assumption for that model.

**Table 8:** OLS model of the Cumulative Response Score.

	Cumulative Response Score
Intercept	9.023 *** [8.018, 10.028]
Food insecurity raw score	0.159 *** [0.052, 0.266]
Transportation burden	0.121 [−0.107, 0.349]
Storage constraints	0.320 *** [0.143, 0.496]
Current food waste	0.391 [−0.145, 0.927]
Shopping frequency	0.117 [−0.143, 0.376]
Household size	−0.308 ** [−0.548, −0.069]
Children in household	0.746 * [−0.003, 1.496]
Any assistance participation	−0.221 [−0.749, 0.308]
Disability	0.856 *** [0.212, 1.501]
Observations	473

Notes: Entries are OLS coefficients with 95% confidence intervals. * p<0.10, ** p<0.05, *** p<0.01.

**Table 9:** OLS channel-specific models.

	Q1 Price	Q2 Carry	Q3 Storage	Q4 Spoilage
Food insecurity raw score	0.057 *** [0.017, 0.096]	0.040 * [−0.004, 0.083]	0.032 [−0.012, 0.075]	0.031 [−0.010, 0.072]
Transportation burden	−0.016 [−0.099, 0.068]	0.114 ** [0.021, 0.207]	0.045 [−0.047, 0.138]	−0.022 [−0.110, 0.065]
Storage constraints	0.044 [−0.021, 0.108]	0.060 [−0.012, 0.132]	0.154 *** [0.082, 0.225]	0.062 * [−0.005, 0.130]
Current food waste	0.032 [−0.164, 0.229]	0.265 ** [0.047, 0.483]	0.094 [−0.124, 0.311]	0.000 [−0.205, 0.205]
Shopping frequency	0.038 [−0.057, 0.133]	−0.042 [−0.148, 0.063]	−0.012 [−0.117, 0.093]	0.133 *** [0.034, 0.233]
Household size	−0.011 [−0.099, 0.076]	−0.097 * [−0.194, 0.001]	−0.146 *** [−0.243, −0.049]	−0.055 [−0.146, 0.037]
Children in household	−0.043 [−0.318, 0.232]	0.236 [−0.069, 0.541]	0.331 ** [0.028, 0.635]	0.222 [−0.065, 0.509]
Any assistance participation	−0.021 [−0.214, 0.173]	−0.106 [−0.321, 0.109]	−0.018 [−0.232, 0.196]	−0.076 [−0.278, 0.126]
Disability	0.153 [−0.083, 0.389]	0.296 ** [0.033, 0.558]	0.172 [−0.089, 0.433]	0.236 * [−0.011, 0.482]
Observations	473	473	473	473

Notes: Entries are OLS coefficients with 95% confidence intervals. These models are provided as supplementary, easier-to-interpret linear specifications corresponding to the ordered-response outcomes. * p<0.10, ** p<0.05, *** p<0.01.

**Table 10:** Summary of research question and hypotheses.

	Construct Tested	Test/Model	Key Result	Verdict
RQ1	Price difference between flexible plastic and alternative packaging formats	Descriptive comparison of means/medians (Approach 1)	Alternative formats higher on average (mean ratio 1.82, median 1.19); sensitive to outliers and clustering	Descriptive; not formally tested
H1	Food insecurity → more severe price-related response severity	Ordered logit, Table 6	OR = 1.10, p<0.05	Supported
H2	Transportation burden → more severe carry-related response severity	Ordered logit, Table 6	OR = 1.23, p<0.05	Supported
H3	Storage constraints → more severe storage-related response severity	Ordered logit, Table 6	OR = 1.31, p<0.01	Supported
H4	Shopping frequency and storage constraints → more severe spoilage-related response severity	Ordered logit, Table 6	Shopping frequency OR = 1.29, p<0.01; storage constraints not significant	Partially supported

Notes: Interaction effects between food insecurity and transportation burden (carry scenario) and between food insecurity and storage constraints (spoilage scenario) were also estimated and were not statistically significant in either case.

## Data Availability

The original contributions presented in this study are included in the article/Supplementary Materials. Further inquiries can be directed to the corresponding author.

## Supplementary Materials

The following supporting information can be downloaded at https://www.mdpi.com/article/10.3390/foods15183188/s1, Figure S1: Additional paired observations from the online and in-store audit (20 September 2025, to 20 October 2025); Note S1: Survey Instrument; Table S1: Response distributions to packaging-change scenarios by food security status; Table S2: Spearman correlation matrix of key constructs; Table S3: Key binary-logistic sensitivity results for outcomes with proportional-odds violations; Table S4: Interaction models between food insecurity and logistical constraints.

## Abbreviations

The following abbreviations are used in this manuscript:

FLW	Food Loss and Waste
EPR	Extended Producer Responsibility
IRB	Institutional Review Board
USDA	United States Department of Agriculture
