# Supplementary material for: The Hidden Costs of Shifting Away from Flexible Packaging: Implications for Food Prices, Access, and Food Waste Among Low-Income U.S. Households
Source: Foods. 2026 Sep 9;15(18):3188. doi: 10.3390/foods15183188 (PMC13605975; doi:10.3390/foods15183188)
Supplement: Supplementary file 1 [file foods-15-03188-s001.zip › foods-4513586-supplementary.pdf]

Supplementary Materials: The Hidden Costs of Shifting Away from Flexible Packaging: Implications for Food Prices, Access, and Food Waste Among Low-Income U.S. Households

Monireh Mahmoudi [1,*], Lauren Davis [2], Natalie Gizoni [1], Sophia Joseph [1] and Kaitlyn Volpi [1]

Supplementary Materials

S1. Figures

Pair No.	Food Category	Food Item	Alternative Packaging	Brand	Weight	Store Name	Online (Y/N)	State	Zipcode	Price ($)	Alternative Packaging	Brand	Weight	Store Name	Online (Y/N)	State	Zipcode	Price ($)
46	Dairy	Parmesan Cheese	Stand Up Pouch	Kraft	6 oz	Walmart	Y	N/A	N/A	4.98	Plastic Cansiter	Kraft	8 oz	Walmart	Y	N/A	N/A	4.44
47	Dry Goods	Rice	Stand Up Pouch	Uncle Ben's	8.8 oz	Walmart	Y	N/A	N/A	2.92	Cups	Minute	2.4 oz	Walmart	Y	N/A	N/A	1.96
48	Dry Goods	Brown Sugar	Lay Down Flat Fin Seal Pouch	Domino	2 lb	Walmart	Y	N/A	N/A	3.67	Paperboard Carton	Domino	16 oz	Walmart	Y	N/A	N/A	8.88
49	Cereals	Frosted Flakes	Lay Down Flat Fin Seal Pouch	Kelogg's	39.5 oz	Walmart	Y	N/A	N/A	6.86	Paperboard Carton	Kellogg's	21.7 oz	Walmart	Y	N/A	N/A	4.98
50	Cereals	Cheerios	Lay Down Flat Fin Seal Pouch	General Mills	32 oz	Walmart	Y	N/A	N/A	6.77	Paperboard Carton	General Mills	27.2 oz	Walmart	Y	N/A	N/A	4.93
51	Cereals	Corn Pops	Lay Down Flat Fin Seal Pouch	Kelogg's	33 oz	Walmart	Y	N/A	N/A	6.86	Paperboard Carton	Kellogg's	8.8 oz	Walmart	Y	N/A	N/A	5.8
52	Cereals	Cinnamon Toast Crunch	Lay Down Flat Fin Seal Pouch	General Mills	32 oz	Kroger	Y	N/A	N/A	7.99	Paperboard Carton	General Mills	12 oz	Kroger	Y	N/A	N/A	4.29
53	Cereals	Fruity Pebbles	Lay Down Flat Fin Seal Pouch	Post	32 oz	Kroger	Y	N/A	N/A	7.99	Paperboard Carton	Post	11 oz	Walmart	Y	N/A	N/A	3.99
54	Infant Foods	Baby Food	Stand Up Pouch	Gerber	3.5 oz	Walmart	Y	N/A	N/A	1.28	Glass Jar	Gerber	4 oz	Walmart	Y	N/A	N/A	1.27
55	Infant Foods	Baby Food	Stand Up Pouch	Beech-Nut	3.5 oz	Walmart	Y	N/A	N/A	1.28	Glass Jar	Beech Nut	4 oz	Walmart	Y	N/A	N/A	0.98
56	Infant Foods	Baby Food	Stand Up Pouch	Parent's Choice	4 oz	Walmart	Y	N/A	N/A	0.87	Plastic Cups	Parent's Choice	8 oz	Walmart	Y	N/A	N/A	1.47
57	Juice	Fruit Juice	Stand Up Pouch	Capri Sun	10 ct 6 fl oz	Walmart	Y	N/A	N/A	3.47	Bottle	Capri Sun	12 oz	Walmart	Y	N/A	N/A	1.62

Figure S1. Additional paired observations from the online and in-store audit (September 20, 2025, to October 20, 2025).

Note S1. Survey Instrument

You are invited to take part in a research survey about access to basic foods and shopping experiences. The survey includes approximately 20 questions and takes about 5 minutes to complete. Participation is voluntary, and you may stop at any time without penalty. You must be 18 years or older to participate. No identifying information will be collected, and responses will remain confidential.

Survey data will be stored on password-protected, MSU-administered cloud storage and accessed only by the research team. You will receive compensation through Prolific after completing the survey and entering the completion code correctly.

If you have questions, please contact Dr. Monireh Mahmoudi (mahmou18@msu.edu). For concerns about participant rights, contact MSU HRPP at irb@msu.edu.

By selecting "I agree," you consent to participate.

- ☐ I agree
- ☐ I do not agree

Q0 Please enter your 5-digit ZIP code. _____

Hypothetical Packaging Changes

For the following questions, please think about a basic food your household buys regularly (for example: milk, fresh produce, meat, or grains).

Q1 If changes in packaging made this food more expensive, what would your household be most likely to do?

- ☐ Continue buying the same amount without difficulty
- ☐ Continue buying it, but reduce spending on other foods or necessities
- ☐ Buy smaller quantities or buy it less often
- ☐ Switch to a cheaper or lower-quality alternative
- ☐ Stop buying this food altogether

Q2 If changes in packaging made this food harder to carry home (for example, heavier or bulkier packaging), what would your household be most likely to do?

☐ No change; we would continue buying it as usual
☐ Buy it less often or in smaller amounts
☐ Rely on someone else or make special trips to get it
☐ Switch to a food that is easier to carry, even if it is less preferred
☐ Stop buying this food altogether

Q3 If changes in packaging made this food harder to store at home (for example, larger size or inefficient shape), what would your household be most likely to do?

☐ No change; storage would not be a problem
☐ Rearrange storage or discard other foods to make space
☐ Buy smaller quantities or shop more frequently
☐ Avoid buying this food unless absolutely necessary
☐ Stop buying this food altogether

Q4 If changes in packaging caused this food to spoil faster, what would your household be most likely to do?

☐ No change; spoilage would not be a concern
☐ Try to use it faster or change meal plans
☐ Buy smaller quantities to avoid waste
☐ Switch to a more shelf-stable or less healthy alternative
☐ Stop buying this food altogether

Accessibility of Grocery Stores

Q5 What is your main way of getting to where you shop for groceries?

☐ My own car
☐ Someone else drives me
☐ Public transit
☐ Walking
☐ Rideshare/taxi
☐ Other

Q6 About how long does it usually take you to get to your main grocery store (one way)?

☐ Less than 10 minutes
☐ 10–20 minutes
☐ 21–40 minutes
☐ 41+ minutes
☐ I don’t have a regular grocery store

Q7 How often do you usually shop for groceries?

☐ More than once per week
☐ Weekly
☐ Every 2 weeks
☐ Monthly

☐ No regular schedule

Storage Capacity

Q8 How would you describe your refrigerator/freezer space at home?

☐ More than enough
☐ Enough
☐ Somewhat limited
☐ Very limited

Q9 How would you describe your pantry/cabinet storage space at home?

☐ More than enough
☐ Enough
☐ Somewhat limited
☐ Very limited

Food Waste

Q10 Thinking about the food your household buys, how much of it typically goes uneaten or is thrown away?

☐ Almost none
☐ A small amount
☐ A moderate amount
☐ A large amount
☐ Almost all of it

Household Food Security Status
For the following statements, please indicate whether each statement was *often true*, *sometimes true*, or *never true* for you or your household during the last 12 months.

Q11 “The food that (I/we) bought just didn’t last, and (I/we) didn’t have money to get more.” Was that often, sometimes, or never true for you or your household in the last 12 months?

☐ Often true
☐ Sometimes true
☐ Never true

Q12 “(I/we) couldn’t afford to eat balanced meals.” Was that often, sometimes, or never true for you or your household in the last 12 months?

☐ Often true
☐ Sometimes true
☐ Never true

Q13 In the last 12 months, did you or other adults in your household ever cut the size of your meals or skip meals because there wasn’t enough money for food?

☐ Yes
☐ No

Q14 If your answer to Q13 was "Yes", how often did this happen?

☐ Almost every month
☐ Some months but not every month
☐ Only 1 or 2 months

Q15 In the last 12 months, did you ever eat less than you felt you should because there wasn't enough money for food?

☐ Yes
☐ No

Q16 In the last 12 months, were you ever hungry but did not eat because there wasn't enough money for food?

☐ Yes
☐ No

Household Characteristics

Q17 How many people live in your household?

☐ 1
☐ 2
☐ 3
☐ 4
☐ 5+

Q18 Do you have children under 18 in your household?

☐ Yes
☐ No

Q19 Do you currently participate in any of the following programs? (Select all that apply.)

☐ SNAP
☐ WIC
☐ School meals (free/reduced)
☐ Food pantry or food bank
☐ Medicaid
☐ None of the above

Q20 Does the primary food shopper or food preparer in your household have a disability that affects shopping, cooking, or food storage?

☐ Yes
☐ No

S. Tables

Table S1. Response distributions to packaging-change scenarios by food security status

Response category	Overall	High/Marginal	Low	Very Low
**Price increase**				
Continue buying the same amount without difficulty	36 (7.6%)	29 (12.3%)	4 (4.0%)	3 (2.2%)
Continue buying it, but reduce spending on other foods or necessities	76 (16.1%)	39 (16.5%)	17 (16.8%)	20 (14.7%)
Buy smaller quantities or buy it less often	131 (27.7%)	64 (27.1%)	30 (29.7%)	37 (27.2%)
Switch to a cheaper or lower-quality alternative	224 (47.4%)	101 (42.8%)	48 (47.5%)	75 (55.1%)
Stop buying this food altogether	6 (1.3%)	3 (1.3%)	2 (2.0%)	1 (0.7%)
**Harder to carry**				
No change; we would continue buying it as usual	261 (55.2%)	148 (62.7%)	47 (46.5%)	66 (48.5%)
Buy it less often or in smaller amounts	106 (22.4%)	48 (20.3%)	25 (24.8%)	33 (24.3%)
Rely on someone else or make special trips to get it	38 (8.0%)	18 (7.6%)	6 (5.9%)	14 (10.3%)
Switch to a food that is easier to carry, even if it is less preferred	64 (13.5%)	22 (9.3%)	21 (20.8%)	21 (15.4%)
Stop buying this food altogether	4 (0.8%)	0 (0.0%)	2 (2.0%)	2 (1.5%)
**Harder to store**				
No change; storage would not be a problem	112 (23.7%)	68 (28.8%)	16 (15.8%)	28 (20.6%)
Rearrange storage or discard other foods to make space	141 (29.8%)	73 (30.9%)	26 (25.7%)	42 (30.9%)
Buy smaller quantities or shop more frequently	144 (30.4%)	71 (30.1%)	32 (31.7%)	41 (30.1%)
Avoid buying this food unless absolutely necessary	54 (11.4%)	19 (8.1%)	17 (16.8%)	18 (13.2%)
Stop buying this food altogether	22 (4.7%)	5 (2.1%)	10 (9.9%)	7 (5.1%)
**Faster spoilage**				
No change; spoilage would not be a concern	23 (4.9%)	15 (6.4%)	2 (2.0%)	6 (4.4%)
Try to use it faster or change meal plans	116 (24.5%)	57 (24.2%)	22 (21.8%)	37 (27.2%)
Buy smaller quantities to avoid waste	218 (46.1%)	125 (53.0%)	43 (42.6%)	50 (36.8%)
Switch to a more shelf-stable or less healthy alternative	58 (12.3%)	18 (7.6%)	14 (13.9%)	26 (19.1%)
Stop buying this food altogether	58 (12.3%)	21 (8.9%)	20 (19.8%)	17 (12.5%)

Table S2. Spearman correlation matrix of key constructs

	Packaging Index	Food Insecurity	Storage	Transport	Shop Freq.	Disability
Packaging Index	1.00	0.21	0.19	0.11	0.06	0.17
Food Insecurity	0.21	1.00	0.16	0.11	0.02	0.18
Storage Constraints	0.19	0.16	1.00	0.05	0.02	0.08
Transportation Burden	0.11	0.11	0.05	1.00	-0.08	0.19
Shopping Frequency	0.06	0.02	0.02	-0.08	1.00	0.13
Disability	0.17	0.18	0.08	0.19	0.13	1.00

Notes: Shop Freq. denotes the shopping-frequency score. Higher values of the Packaging Index indicate greater packaging-related accessibility burden.

Table S3. Key binary-logistic sensitivity results for outcomes with proportional-odds violations

Outcome	Binary specification	Focal predictor	OR	95% CI	p
Q1 Price	No change ($n = 36$) vs. any reported change ($n = 437$)	Food insecurity score	1.48	[1.17, 1.86]	0.001
Q4 Spoilage	Milder response ($n = 357$) vs. severe response ($n = 116$)	Shopping frequency	1.47	[1.17, 1.83]	0.001

Notes: The table reports the focal robustness result for each outcome for which the proportional-odds assumption was not supported. For Q1, the binary outcome contrasts no reported change with any reported change. For Q4, the severe-response category combines switching to a more shelf-stable or less healthy alternative and stopping purchase altogether; all less severe responses form the reference category. A conventional Q4 collapse of no change ($n = 23$) versus any change ($n = 450$) was also estimated for transparency; because the no-change category is very small, estimates from that specification are imprecise. In that conventional collapse, none of the predictors reached statistical significance: food insecurity OR=1.01 ($p = 0.893$), transportation burden OR=0.84 ($p = 0.716$), storage constraints OR=1.63 ($p = 0.288$), disability OR=2.20 ($p = 0.308$), assistance participation OR=1.45 ($p = 0.442$), shopping frequency OR=0.90 ($p = 0.630$), household size OR=0.89 ($p = 0.554$), and children in household OR=0.84 ($p = 0.786$).

Table S4. Interaction models between food insecurity and logistical constraints

	Q2 Harder to Carry Food Insecurity × Transportation	Q4 Faster Spoilage Food Insecurity × Storage
Food insecurity raw score	1.06 [0.95, 1.18]	1.04 [0.89, 1.20]
Transportation burden	1.19 [0.93, 1.53]	0.96 [0.82, 1.13]
Storage constraints	1.16** [1.02, 1.32]	1.10 [0.92, 1.33]
Current food waste	1.63** [1.11, 2.40]	1.04 [0.71, 1.51]
Shopping frequency	0.92 [0.76, 1.10]	1.29*** [1.07, 1.55]
Household size	0.85* [0.71, 1.02]	0.92 [0.78, 1.09]
Children in household	1.23 [0.70, 2.15]	1.37 [0.80, 2.35]
Any assistance participation	0.82 [0.55, 1.20]	0.89 [0.62, 1.29]
Disability	1.68** [1.07, 2.63]	1.46 [0.93, 2.30]
Food insecurity × transportation burden	1.01 [0.95, 1.08]	
Food insecurity × storage constraints		1.01 [0.96, 1.06]
Observations	473	473

Notes: Entries are odds ratios with 95% confidence intervals from ordered logit interaction models. * $p < 0.10$, ** $p < 0.05$, *** $p < 0.01$. Interaction terms were estimated selectively for scenarios where clear theoretical complementarities were expected. Specifically, interactions between food insecurity and transportation burden were examined for the harder-to-carry scenario, and interactions between food insecurity and storage constraints were examined for the faster-spoilage scenario. These specifications reflect the underlying mechanisms through which logistical constraints may amplify accessibility challenges. No interactions were estimated for the price increase scenario, as this channel primarily reflects direct financial constraints, nor for the storage scenario, where storage limitations already capture the primary mechanism.
